# Oxaliplatin related lncRNAs prognostic models predict the prognosis of patients given oxaliplatin-based chemotherapy

**DOI:** 10.1186/s12935-023-02945-3

**Published:** 2023-05-27

**Authors:** Qing-nan Zhou, Rong-e Lei, Yun-xiao Liang, Si-qi Li, Xian-wen Guo, Bang-li Hu

**Affiliations:** 1 Department of Gastroenterology, The People’s Hospital of Guangxi Zhuang Autonomous Region & Research center of Gastroenterology, Guangxi Academy of Medical Sciences, No. 6 Taoyuan Road, Nanning, 530021 Guangxi China; 2 Department of Gastroenterology, The First Affiliated Hospital of Guangxi Medical University, Nanning, 530021 Guangxi China; 3 Department of Research, Guangxi Medical University Cancer Hospital, No. 71 Hedi Road, Nanning, 530021 Guangxi China

**Keywords:** Cancer, Oxaliplatin sensitivity, lncRNAs, Machine learning algorithm

## Abstract

**Background:**

Oxaliplatin-based chemotherapy is the first-line treatment for colorectal cancer (CRC). Long noncoding RNAs (lncRNAs) have been implicated in chemotherapy sensitivity. This study aimed to identify lncRNAs related to oxaliplatin sensitivity and predict the prognosis of CRC patients underwent oxaliplatin-based chemotherapy.

**Methods:**

Data from the Genomics of Drug Sensitivity in Cancer (GDSC) was used to screen for lncRNAs related to oxaliplatin sensitivity. Four machine learning algorithms (LASSO, Decision tree, Random-forest, and support vector machine) were applied to identify the key lncRNAs. A predictive model for oxaliplatin sensitivity and a prognostic model based on key lncRNAs were established. The published datasets, and cell experiments were used to verify the predictive value.

**Results:**

A total of 805 tumor cell lines from GDSC were divided into oxaliplatin sensitive (top 1/3) and resistant (bottom 1/3) groups based on their IC50 values, and 113 lncRNAs, which were differentially expressed between the two groups, were selected and incorporated into four machine learning algorithms, and seven key lncRNAs were identified. The predictive model exhibited good predictions for oxaliplatin sensitivity. The prognostic model exhibited high performance in patients with CRC who underwent oxaliplatin-based chemotherapies. Four lncRNAs, including C20orf197, UCA1, MIR17HG, and MIR22HG, displayed consistent responses to oxaliplatin treatment in the validation analysis.

**Conclusion:**

Certain lncRNAs were associated with oxaliplatin sensitivity and predicted the response to oxaliplatin treatment. The prognostic models established based on the key lncRNAs could predict the prognosis of patients given oxaliplatin-based chemotherapy.

**Supplementary Information:**

The online version contains supplementary material available at 10.1186/s12935-023-02945-3.

## Background

Oxaliplatin, a third-generation platinum anti-cancer medicine, is a cytotoxic chemotherapeutic agent that kills tumor cells by impeding the synthesis of RNA, DNA, and protein in those cells [1]. In current clinical setting, oxaliplatin is used to treat colorectal cancer (CRC) as adjuvant treatment of stage III CRC patients who have undergone surgery resection of primary tumor [2]. Besides CRC, oxaliplatin also used for the treatment of pancreatic cancers [3] and is undergoing clinical trials in ovarian cancer [4], gastric cancer [5], and lung cancer [6]. For human beings, there were several types of cancers, which contain diverse tumor cells with distinctly properties, but the effect of oxaliplatin on these cancers is varied greatly. Therefore, finding the tumor cells that sensitive to oxaliplatin treatment is imperative for the administration of oxaliplatin in those cancers.

At present, studies have shown that several molecular are capable to distinguish whether the cancers are sensitive to oxaliplatin-based chemotherapies. Lai et al. [7] constructed a three DNA repair-related genes signature (RECQL, POLQ, and RAD17) to predict the prognosis of pancreatic cancer, and RAD17 expression is correlated with oxaliplatin treatment. We previous using gene profiles datasets to establish predictive model for the CRC prognosis, and constructed a oxaliplatin related-gene model and showed good predictive value on CRC prognosis [8]. In addition, POU2F1 [9], FXYD6 [10] and DDB2 [11] are reported to associate with the prognosis of patients with different cancers underwent oxaliplatin-based chemotherapies. These evidences demonstrated that some genes and the signatures are reliable biomarkers to distinguish cancers that sensitive to oxaliplatin-based chemotherapies.

Long noncoding RNAs (lncRNAs), a group of transcripts with a length of more than 200 nucleotides, are important for controlling cellular functions and preserving cell health. lncRNAs have emerged as candidate tools for therapeutic intervention and prognosis prediction [12, 13]. Several lncRNAs have been implicated in the oxaliplatin sensitivity of cancers. For instance, silenced LINC01134 enhances oxaliplatin sensitivity in hepatocarcinoma [14], LINC00963 regulates the suppress effect of oxaliplatin in gastric cancer via targeting ATG16L1 expression [15], LINC00525 knockdown increased sensitivities to oxaliplatin in CRC [16]. However, most of the current evidence only focus on the biological function of lncRNAs with individual cancer, the association of lncRNAs with oxaliplatin sensitivity in multiple cancers remains needs to explore. In this study, we screen lncRNAs that related to oxaliplatin sensitivity from multiple tumor cells, and constructed predictive model using machine learning algorithm, then determine the association with patient prognosis in several cancers, finally, we validated the expression of lncRNA in external dataset and cells experiment.

## Materials and methods

### Data acquisition and processing

The data of tumor cell lines treatment with oxaliplatin was downloaded from Genomics of Drug Sensitivity in Cancer (GDSC) database (www.sanger.ac.uk/) [17], including the name of cell lines, IC50 value and transcripts express profile of each cell line after oxaliplatin treatment. lncRNAs was extracted from the transcripts based on Homo sapiens GRCh38 file. There were 5710 transcripts were defined as lncRNAs, after mapping to the transcripts ID of GDSC dataset, 1002 lncRNAs were extracted and proceed to the analysis. The RNA-seq dataset of tumor cells and drug information was downloaded from CellMiner database (discover.nci.nih.gov/cellminer/), which contains a panel of 60 diverse human tumor cell lines and over 100,000 chemical compounds and natural products.

The gene expression profile data and the corresponding clinical data of COADREAD patients (colon and rectal adenocarcinoma; 367 samples), LUSC (lung squamous cell carcinoma; 175 samples), LUAD (lung adenocarcinoma; 475 samples), STAD (stomach adenocarcinoma; 440 samples), PAAD (pancreatic adenocarcinoma; 176 samples) and OV (ovarian serous cystadenocarcinoma; 247 samples) were obtained freely from the UCSC Xena database. GSE76092 dataset [18] containing the data of two CRC cell lines treatment with oxaliplatin, was downloaded from GEO database. The raw data of the above datasets were preprocessed via background adjustment and quantile normalization. Differentially expressed analysis for the datasets was used “edgeR” package, with the |logFC| >0.05 and P < 0.05 as selection thresholds.

### Construction of predictive and prognostic model based on lncRNAs

The oxaliplatin sensitivity predictive model was constructed by incorporating the lncRNAs into four machine learning algorithms, that is LASSO, Decision tree (DT), Random forest (RF) and support vector machine (SVM), in which the data of lncRNAs were divided into training set and tested set at the ratio of 7:3. The significant lncRNAs were screened in the training set and then the predictive value was determined in tested set by using ROC and AUC analysis. lncRNAs identified from the machine learning algorithms were overlapped to obtain common lncRNAs, which was defined as key lncRNAs. Afterwards, logistic regression analysis was conducted to verify the predictive value based on the key lncRNAs.

The prognostic lncRNAs model for the patients was established by Cox regression analysis and characterized by the risk score as previously reported. Based on the previously established formula, the regression coefficient (β) from the multivariate Cox regression model multiplies the expression of the matching lncRNA to determine the risk score [19]. Then the patients were divided into high- and low-risk group using median of risk score as cut-off value. Next, the Kaplan–Meier plot was applied the examine the association with the survival of patients. Finally, the prognostic value of model was determined using ROC and AUC analysis.

### Cells culture and RT-PCR assay

Two CRC cell lines (HCT116 and HT29) were bought from the Shanghai Cell Bank of the Chinese Academy of Sciences and cultured in DMEM supplemented with 10% fetal bovine serum (FBS) and 100 µg/mL of penicillin- streptomycin. Oxaliplatin (Qilu Pharmaceutical Co., Ltd in Jinan, China) was dissolved in DMSO at a concentration of 30 mol/L and the cells were treated by oxaliplatin for 24 h. CCK8 assay kit (Meilun Co.,Ltd, Nanjing, China’s) was used to detect the vitality of the cells according to the manufacturer’s instructions. Briefly, 2 × 10^3^ of cells were plated in 96 well plates. After treatment with DMOS or oxaliplatin, cells were incubated in 10% CCK8 reagent for 2 h. The OD value was measured at 450 nm with TECAN Infinite 200 PRO Plate reader. Total RNA was extracted using the TRIzol chemical. The primary strand DNA was created using the PrimeScript RT chemical agent Kit and gDNA tool (Invitrogen; Thermo Fisher Scientific, Inc.). Step-by-step RT-PCR procedures were carried out in accordance with the manufacturer’s instructions. The expression of lncRNAs was measured using the 2^−ΔΔ^CT method. In Supplemental Tables 1, the primers for the seven lncRNAs used in the RT-PCR test are listed.

### Statistical analysis

The R software was used to conduct each statistical analysis (Version: 4.1.2). For contrasting two groups, the independent Student’s t test or Wilcoxon test was applied to continuous data. The Kaplan-Meier survival analysis and log-rank test were used to assess the overall survival (OS) between two groups. Predictive models were built using logistic or Cox regression analyses. Receiver operating characteristic curves (ROC) was used to visualize the predictive model, and the AUC (area under the ROC curve) was adopted to quantify the predictive value of ROC curve. The Time-dependent ROC was applied to evaluate the predictive value of survival time by “timeROC” package in R. Statistical significance was determined to be a two-tailed P-value 0.05.

## Results

### Screen lncRNA sensitive to the oxaliplatin in tumor cells

**Fig. 1:**
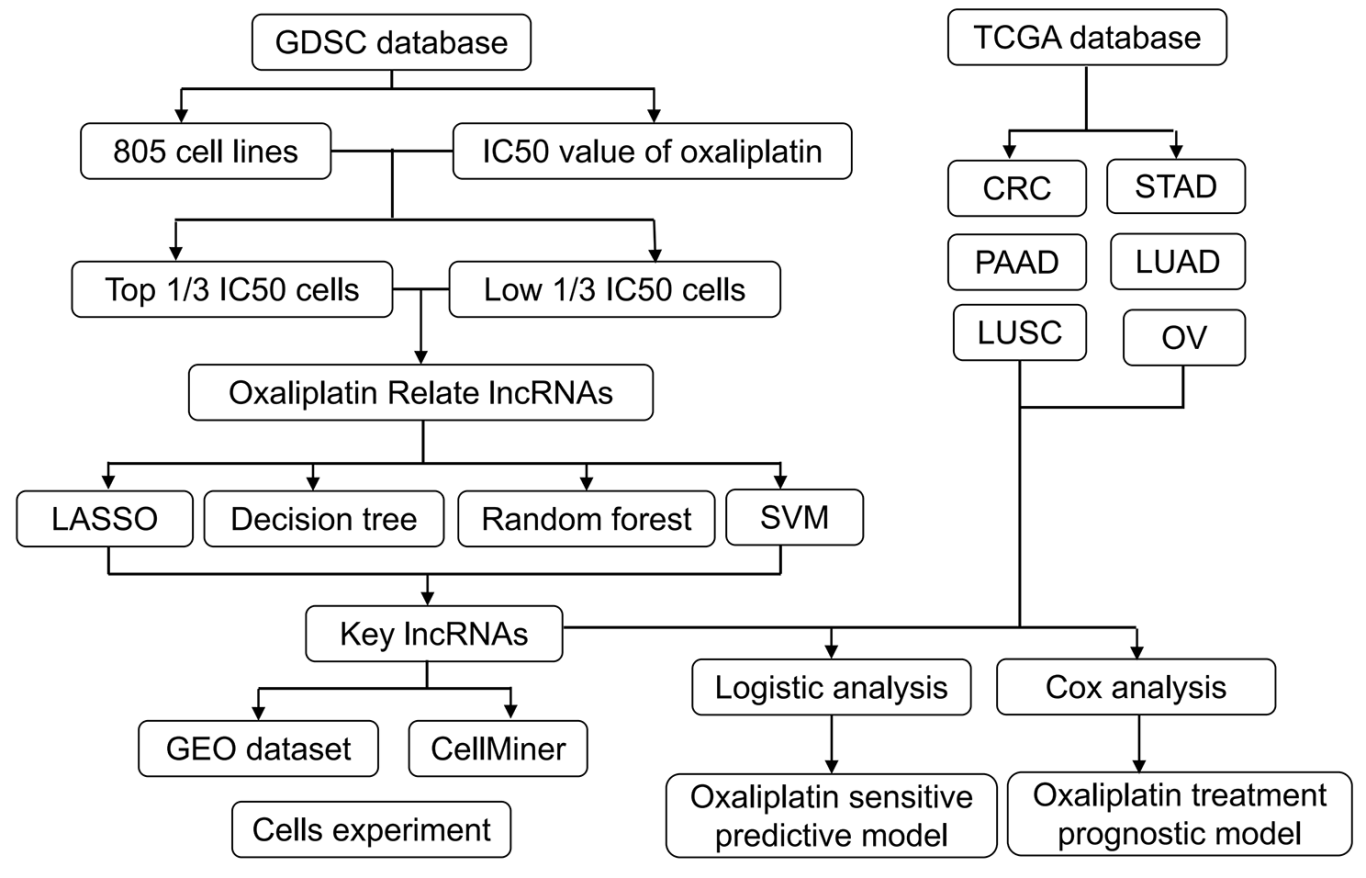
Flow diagram of study design

The flow diagram of this study was summarized in Fig. 1. We firstly selected the data of oxaliplatin treatment in tumor cells from GDSC, which included the name of 805 cells and the IC50 value that treated by oxaliplatin. The cell lines were divided into three groups based on the IC50 value, the top 1/3 (322) with a low IC50 value (IC50 ≤ 20.8) were selected as sensitive group, while the last 1/3 (323) with a low IC50 value (IC50 ≥ 99.3) were enrolled in the resistant group. With the lower IC50 value, the stronger the sensitivity to the oxaliplatin treatment.

**Fig. 2:**
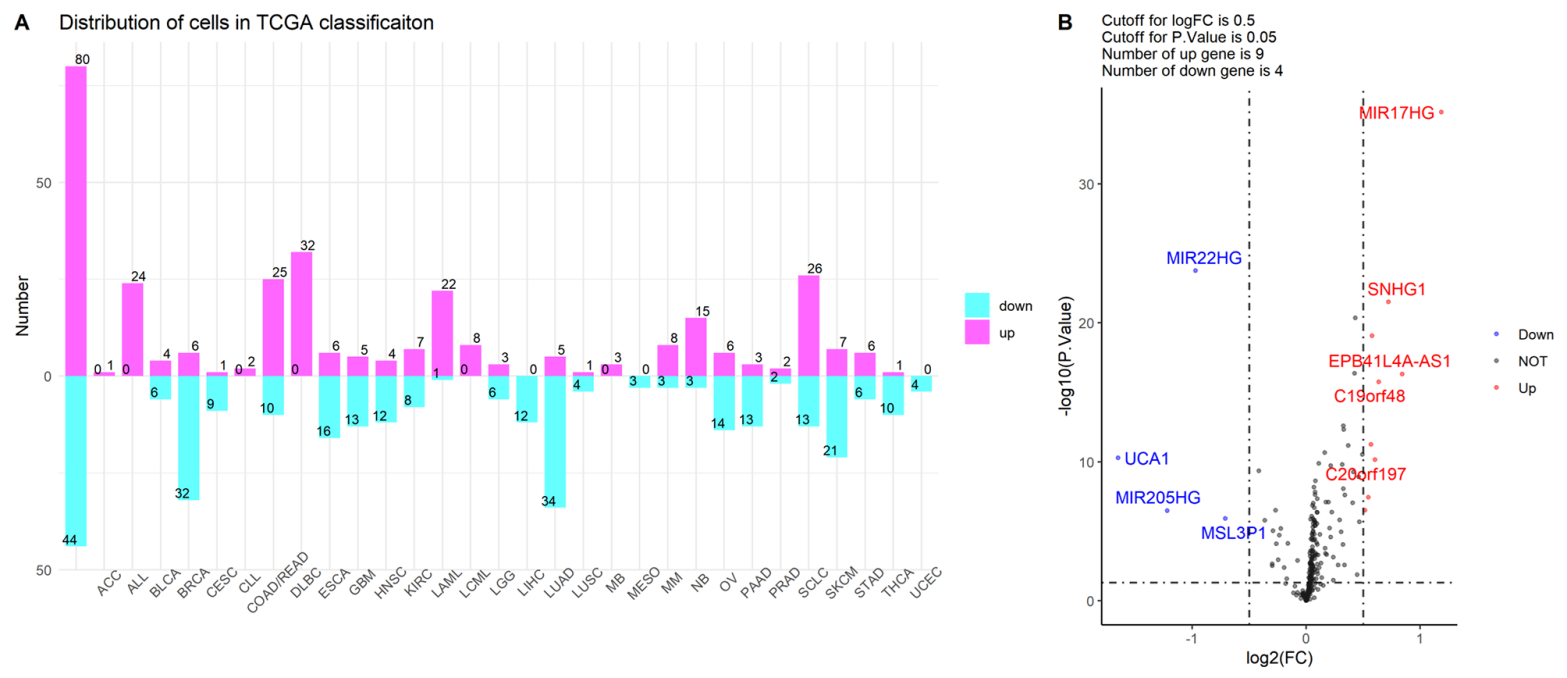
Screen lncRNAs related to the oxaliplatin sensitivity. (**A**) Distribution of tumor cells in TCGA classification; (**B**) Volcano plot of differential expression analysis for lncRNAs.

The distribution of the cell lines in TCGA classification was listed in Fig. 2A, and the results suggested that all the tumor cells of ALL (acute lymphoblastic leukemia; 22 cells) and CRC (20 cells), and most of the LAML cells (acute myeloid leukemia; 18 cells) showed more sensitivity to the oxaliplatin treatment than other type of tumor cells. Next, the data of lncRNAs were extracted from the dataset, and then differential expression analysis was performed on these two groups (sensitive group and resistant groups). Based on the defined criteria (|logFC| >0.05 and P < 0.05), 13 lncRNAs showed significantly differential expressed in two groups (Fig. 2B).

### Construction of predictive model to the oxaliplatin sensitivity by machine learning algorithm

**Fig. 3:**
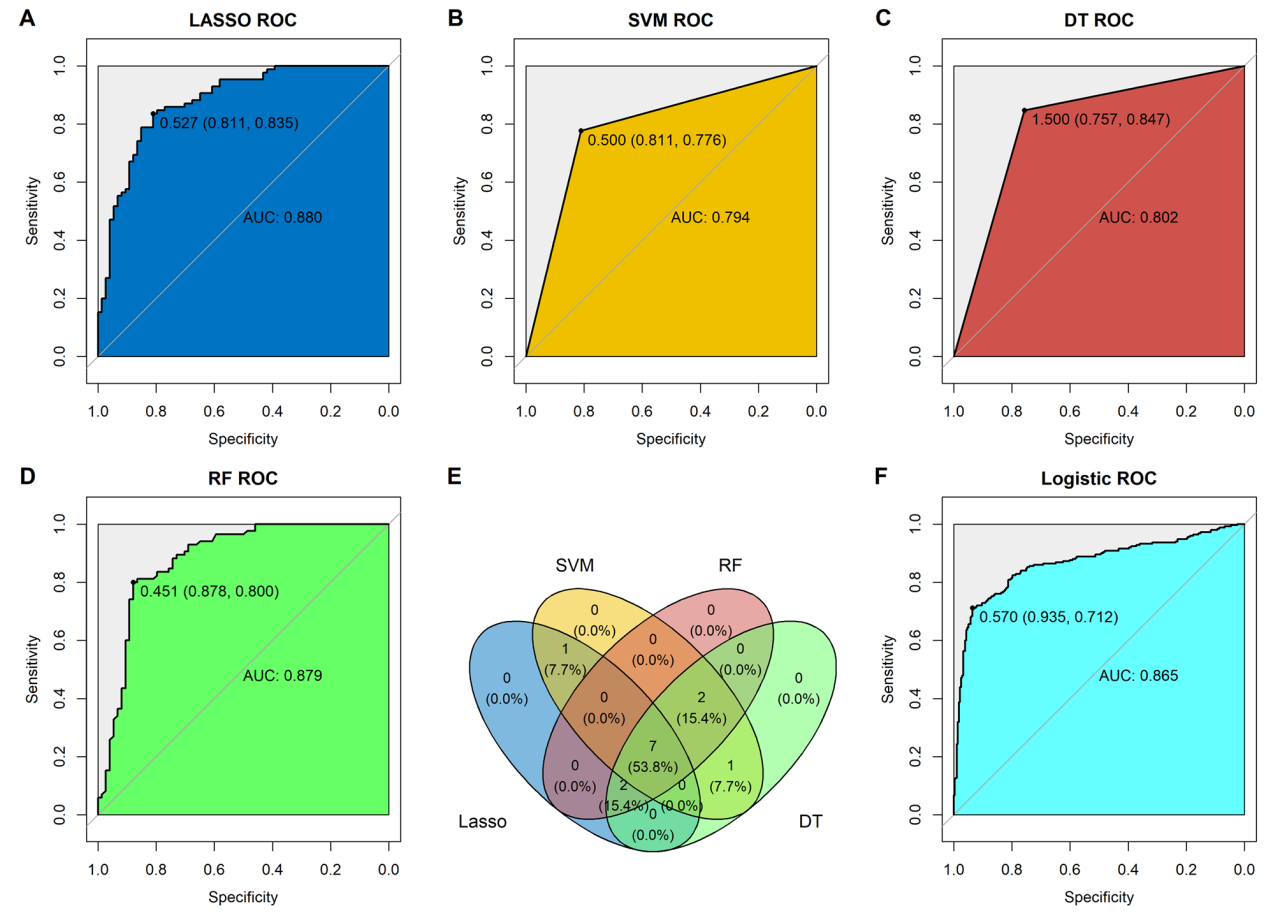
Construction of predictive model to the oxaliplatin sensitivity. Predictive value of (**A**) LASSO algorithm; (**B**) SVM algorithm; (**C**) Decision tree algorithm; (**D**) Random-forest algorithm; (**E**) Venn plot for the lncRNAs from the four machine learning algorithm; (**F**) Predictive value of logistic value base on the 7 lncRNAs.

To construct predictive model to the oxaliplatin sensitivity based on 13 differential expressed lncRNAs, four machine learning algorithm, including LASSO, DT, RF and SVM, were employed by incorporated the lncRNAs. The number of training set and test set was 563 and 242, respectively. The ROC of each model that established by the four machine learning algorithms was built, and all these models showed high predictive value to the oxaliplatin sensitivity, with the AUC value range from 0.794 to 0.880 (Fig. 3A-D). After overlapping the importance lncRNAs based on each model, seven lncRNAs (CMAHP, NSUN5P2, SNHG1, MIR17HG, MIR22HG, C19orf48, UCA1) remain. Then logistic regression algorithm was applied to construct the predictive model using the seven lncRNAs and visualized and estimated by ROC and corresponding AUC (Fig. 3E). Consistent to the models from above four machine learning algorithms, the logistic regression model showed that this model has better predictive value to the oxaliplatin sensitivity, with the AUC value as 0.865 (Fig. 3F).

### Predictive value of lncRNAs in CRC patients’ prognosis underwent oxaliplatin-based chemotherapy

**Fig. 4:**
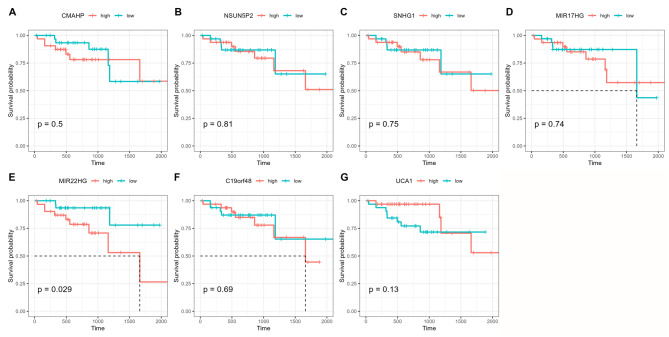
Association of seven lncRNAs with the survival of CRC patients who underwent oxaliplatin treatment. (**A**) CMAHP; (**B**) NSUN5P2; (**C**) SNHG1; (**D**) MIR17HG; (**E**) MIR22HG; (**F**) C19orf48; (**G**) UCA1.

Since oxaliplatin-base chemotherapies was administrated as first line chemotherapy in CRC, we thus explored the prognostic value of lncRNAs in CRC prognosis by analyzing the TCGA-COADREAD dataset. The samples that treated by oxaliplatin or included oxaliplatin as chemotherapy regimen were selected, finally 70 samples were collected. However, only five of the seven lncRNAs were found in the TCGA dataset, hence, we conducted the analysis for these five lncRNAs. As the results showed, using median value as cut-off, only MIR22HG was significantly related to the survival of CRC patients who received the oxaliplatin-based treatment, the other six lncRNAs showed little association (Fig. 4).

**Fig. 5:**
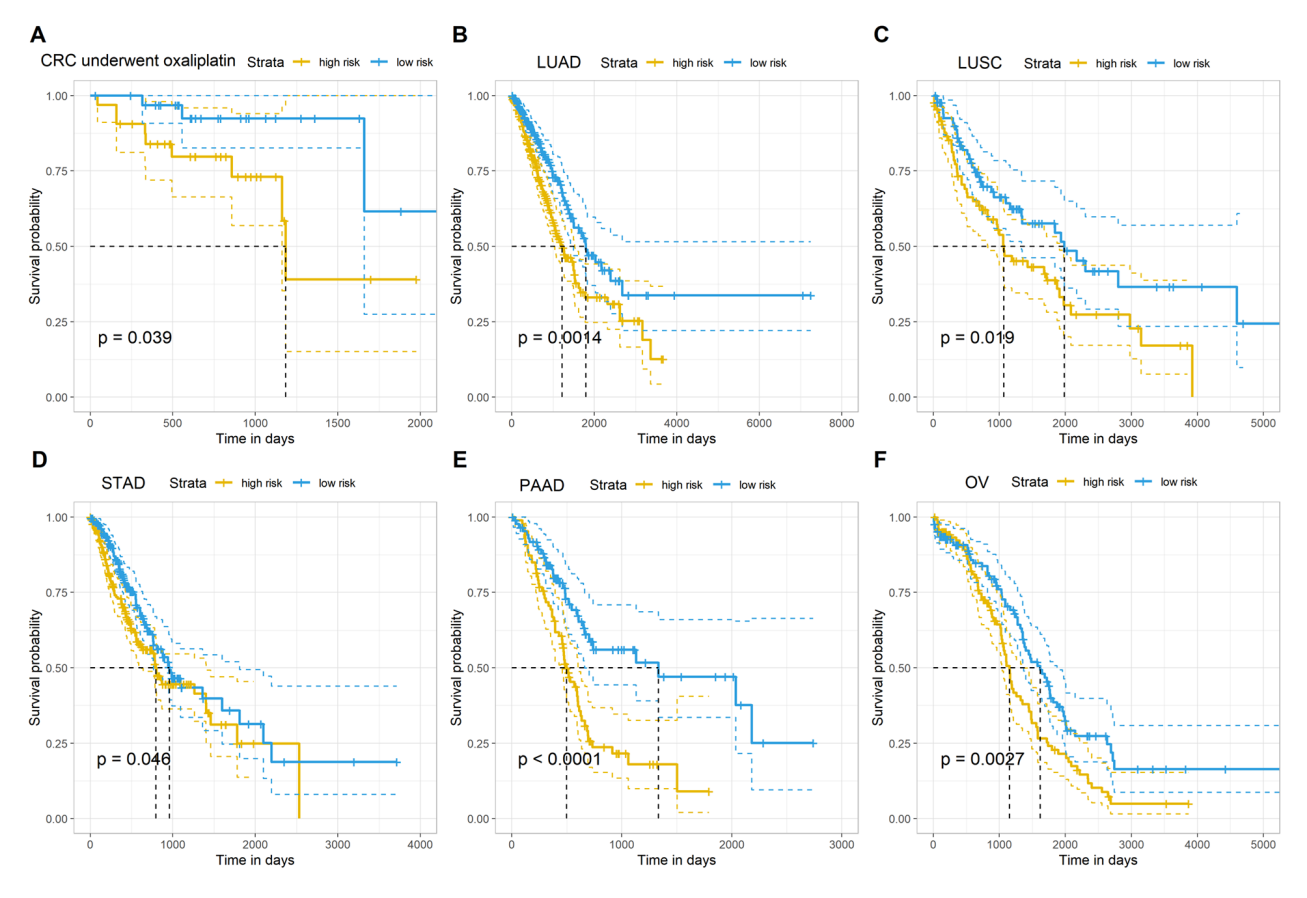
The association of risk score with patients’ survival in (**A**) CRC patients underwent oxaliplatin-base chemotherapies; (**B**) LUAD; (**C**) LUSC; (**D**) STAD; (**E**) PAAD; (**F**) OV.

**Fig. 6:**
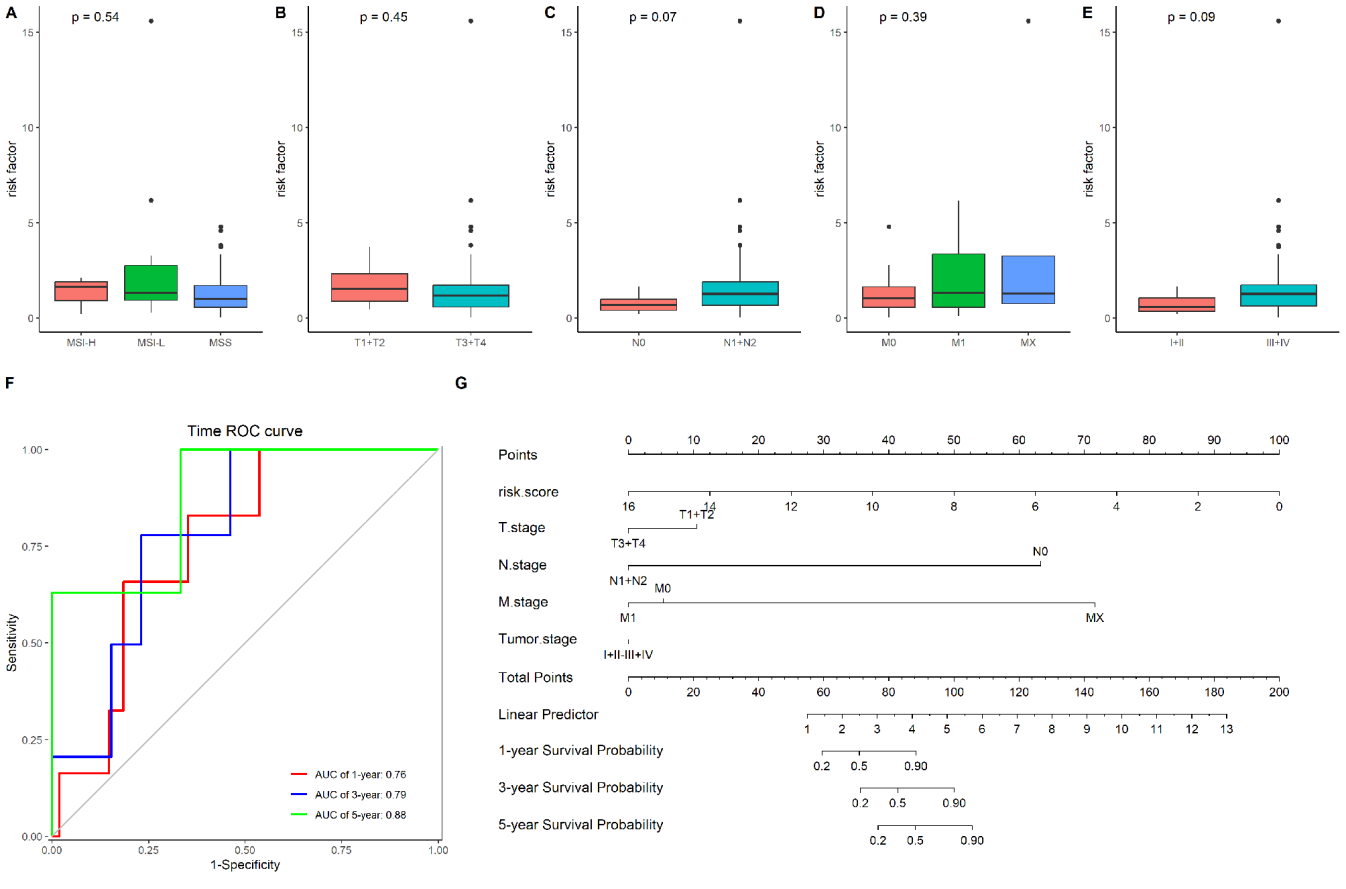
Association of risk score with CRC patients underwent oxaliplatin-base chemotherapies in (**A**) MSI status; (**B**) T stage; (**C**) N stage; (**D**) M stage; (**E**) Tumor stage; (**F**) Time-dependent ROC of the risk score for the survival of CRC patients; (**G**) Nomogram graph for the risk score and TNM stage and Tumor stage in prognostic patients’ survival

Then, using the Cox regression algorithm and a risk score calculation, we built a model using these five lncRNAs. We could see that CRC patients with high-risk scores have a worse prognosis than those with low-risk scores by dividing the samples into high- and low-risk groups based on the median value of the risk score (Fig. 5A), indicating the high predictive value of the model for CRC patients who received oxaliplatin-based chemotherapies. We also constructed prognostic model for LUSC, LUAD, STAD, PAAD, and OV, which were reported to has certain response to oxaliplatin treatment, and found that the model based on the lncRNAs has good performance to the prognosis for these cancers (Fig. 5B-F). Next, we determined the association of the risk score with the clinical features in CRC. As Fig. 6A-E showed, the risk score was not associated with the MSI status, TNM stage and tumor stage. Then we used Time-dependent ROC and the corresponding AUC to estimate the prognostic value of the risk score, and found this risk score has better prognostic ability to the survival of patients, with the AUC value of 1-, 3-, 5-years survival as 0.76, 0.79 and 0.88, respectively (Fig. 6F). Finally, we used Nomogram graph to visual the prognostic value of risk score, and the results showed that risk score has better prognostic value than TNM stage and tumor stage in CRC patients underwent oxaliplatin-base chemotherapies (Fig. 6G).

### Validated the expression of lncRNAs in CRC cells treatment with oxaliplatin

**Fig. 7:**
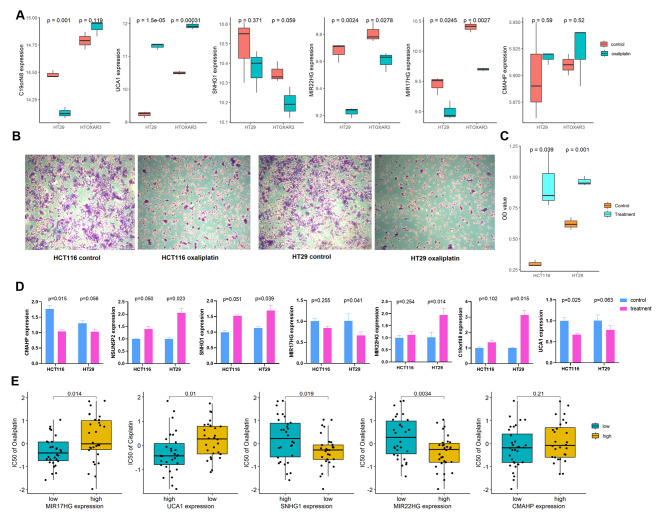
Validated the expression of lncRNAs in CRC cells treatment with oxaliplatin (**A**) Expression of lncRNAs in GSE76092 dataset validation; (**B**-**C**) CCK8 showed the proliferation and OD value of HCT116 and HT29 cells treatment with oxaliplatin; (**D**) Expression of lncRNAs in CRC cells treatment with oxaliplatin by RT-PCR assay; (**E**) Expression of lncRNAs in “cellMiner” database

The expression of 7 lncRNAs was firstly verified in an external dataset (GSE76092), which provided the data of two CRC cells (HT29 and HTOXAR3) treated by oxaliplatin at dose of 10 mM. Six lncRNAs were found in the dataset, and the results indicated that, C19orf48, UCA1, MIR22HG, MIR17HG show significant different expression in HT29 cell between with or without oxaliplatin treatment, while UCA1, MIR22HG, MIR17HG showed significant difference in HTOXAR3 cells (Fig. 7A). Next, we tested the expression of 7 lncRNAs using RT-PCR method in two CRC cells (HCT116 and HT29) by treating with oxaliplatin. We observed that oxaliplatin could significantly inhibit proliferation of these two CRC cells (Fig. 7B-C), but only UCA1, MIR22HG, MIR17HG showed significantly different after oxaliplatin treatment in both cell lines (Fig. 7D). Finally, we examined these lncRNAs with oxaliplatin and cisplatin sensitivity by “cellMiner” database, and found that MIR22HG, MIR17HG, SNHG1 was associated with oxaliplatin sensitivity (Fig. 7E). The results showed that all not the lncRNAs present the same response to oxaliplatin treatment in different CRC cells.

## Discussion

Aberrant expression of lncRNAs is a consistent characteristic occur in almost all types of cancers. lncRNAs also show higher tissue and cell subtype specificities than most mRNAs [20], and play their biological roles through the regulation of coding gene expression at the epigenetic, transcriptional, and post-transcriptional levels, subsequently modulate the pathogenesis, progression of cancers [21, 22]. Given their specific expression and functional diversities in a variety of cancers, lncRNAs have received extensive attention regarding their potential applications as diagnostic and prognostic biomarkers and/or therapeutic targets in many kinds of cancers. Chemotherapy is nonsurgical treatment for patients with cancers. However, no reliable precise molecular markers are available to determine which patients can benefit from it. Hence, finding lncRNAs that related to chemotherapy sensitivity could help to select those patients who are suitable for the specific for the chemotherapy.

In the present study, we screened lncRNAs that sensitive to oxaliplatin from multiple types of tumor cells, and found that tumor cells from ALL, CRC, and LAML were oxaliplatin sensitivity. Then the 13 lncRNAs from differential expression analysis were used to construct predictive model for tumor cells that are oxaliplatin sensitivity. The four machine learning algorithms showed that the model constructed base on these lncRNAs has good predictive value, and these machine learning algorithms identified seven significant lncRNAs in the model. Importantly, for CRC patients underwent oxaliplatin-based chemotherapies, the prognostic model based on the key lncRNAs showed high predictive value. Moreover, the predictive model also exhibited excellent performance in the prognosis of LUSC, LUAD, STAD, PAAD, and OV patients. However, the little associations of model with the clinical features suggested this model is independent of the status of CRC, which indicate the reliable of this predictive model.

In the validation analysis, the results from GSE76092 dataset, and our cells experiment failed to confirm the expression of all the seven lncRNAs in CRC cells that treatment with oxaliplatin, only four lncRNAs (C20orf197, UCA1, MIR17HG, and MIR22HG) were consistent in the above two analysis. In addition, the “cellMiner” dataset also failed to verify the sensitivity of seven lncRNAs with oxaliplatin in cancer cells. These results demonstrated the heterogeneity of the tumor cells from different studies. To be noted, among the four lncRNAs, only UCA1 was reported to associated with oxaliplatin sensitivity in hepatocellular carcinoma [23] and CRC [24]. C20orf197 has been showed to associate with the survival of patients with lung adenocarcinoma [25]. MIR17HG and MIR22HG have been wildly investigated in many cancers, including CRC [26, 27], gastric cancer [28, 29], and MIR17HG was also related to the paclitaxel resistance in ovarian cancer [30]. These evidences suggested that the role of seven lncRNAs is oxaliplatin sensitivity still need further validation.

Although our study failed to verify these seven lncRNAs were all oxaliplatin sensitivity in CRC cells, but we confirmed that the model constructed from the lncRNAs has high predictive value in predicting oxaliplatin sensitivity the prognosis of patients underwent oxaliplatin-based chemotherapies. We also identified three lncRNAs that were not investigated their association with oxaliplatin sensitivity, suggesting that future studies could be conducted to determine this association. Nevertheless, there were several limitations in this study. Firstly, the expression of the seven lncRNAs were not tested in more tumor cells, including different types of cancers. Second, the present cell experiment used only one dose of oxaliplatin to treat the CRC cells, but the response to the oxaliplatin treatment is varied in different CRC cells, which might be the reasons that expression some lncRNAs did not change simultaneously. Third, we lack of experiment of silence and overexpression of these lncRNAs in tumor cells, which is necessary to validated the effect of the lncRNAs in tumor cells after oxaliplatin treatment. Therefore, future study is warrant to address these issues.

## Conclusions

The present study identified several lncRNAs that related to oxaliplatin sensitivity using machine learning algorithms, and construct a predictive and prognostic model based on the lncRNAs, which showed good predictive value in oxaliplatin sensitivity and patients underwent oxaliplatin-based chemotherapies. More studies are needed to further confirm the role of these lncRNAs in cancer that treatment with oxaliplatin.

## Electronic supplementary material

Below is the link to the electronic supplementary material.

Supplementary Material 1

## Data Availability

The data used to support the findings of this study are available from the corresponding author upon request.
