# Supplementary material for: Oxaliplatin related lncRNAs prognostic models predict the prognosis of patients given oxaliplatin-based chemotherapy
Source: Cancer Cell Int. 2023 May 27;23:103. doi: 10.1186/s12935-023-02945-3 (PMC10223895; doi:10.1186/s12935-023-02945-3)
Supplement: Supplementary file 1 — Supplementary Material 1 [file 12935_2023_2945_MOESM1_ESM.doc]

**Supplement files**

Table S1 Primer Sequence of genes

| Genes |  | Primer Sequence |
| --- | --- | --- |
| C19orf48 | FORWARD | 5’- AGAAATGCTGGGGTGCAG -3’ |
|  | REVERSE | 5’- AGTAACAGGCAGCCTCCTCTG -3’ |
| UCA1 | FORWARD | 5’- ACGCTAACTGGCACCTTGTT-3’ |
|  | REVERSE | 5’- CTCCGGACTGCTTCAAGTGT-3’ |
| MIR22HG | FORWARD | 5’- CGGACGCAGTGATTTGCT -3’ |
|  | REVERSE | 5’- GCTTTAGCTGGGTCAGGACA -3’ |
| MIR17HG | FORWARD | 5’- TCAGGAGTTCGAGACCAACC -3’ |
|  | REVERSE | 5’- TGCCTCAGCCTCCAGAGTAG -3’ |
| SNHG1 | FORWARD | 5’- GCCAGCACCTTCTCTCTAAAGC -3’ |
|  | REVERSE | 5’- GTCCTCCAAGACAGATTCCATTTT -3’ |
| CMAHP | FORWARD | 5’-CCTTGGCACTGTGGATGCTCA-3’ |
|  | REVERSE | 5’-CTAGTGTTGAGGATACACACTTCC-3’ |
| LINC00158 | FORWARD | 5’-CCAGAGCCATTTTGGAACTTTACC-3’ |
|  | REVERSE | 5’-CCTCCAGTCTTGGTCACTTCCA-3’ |
| GAPDH | FORWARD | 5’-GGACCTCATGGCCTACATGG-3’ |
|  | REVERSE | 5’-TAG GGCCTCTCTTGCTCAGT-3’ |
